# Enhanced PEMFC durability with graphitized carbon black cathode catalyst supports under accelerated stress testing

**DOI:** 10.1039/d1ra01468d

**Published:** 2021-05-28

**Authors:** Qiong Xue, Jian-biao Huang, Dai-jun Yang, Bing Li, Cun-man Zhang

**Affiliations:** Clean Energy Automotive Engineering Center, School of Automotive Studies, Tongji University Shanghai 201804 China yangdaijun@tongji.edu.cn

## Abstract

The anti-corrosion properties of the carbon substrates of cathode catalysts play a vital role in the commercialization of fuel cell vehicles. Our report reveals the enhanced durability of graphitized carbon black catalyst substrates in polymer electrolyte membrane fuel cells (PEMFCs), tested under simulated start-stop cycling and high potential holding conditions. Graphitized carbon treated at various temperatures is used as the support for Pt catalysts. The catalyst utilizing graphitized carbon treated at 1800 °C demonstrates superior antioxidation properties and the inhibition of Pt particle coarsening. The decay ratio of the potential at 1000 mA cm^−2^ has been reduced from 34.9% (commercial Pt/C) to 0.5% during high potential holding accelerated stress testing. Correspondingly, the growth of Pt particles is reduced from 0.95 nm (commercial Pt/C) to 0.08 nm; that is, the coalescence of Pt particles is effectively alleviated upon using graphitized carbon black.

## Abstract 2

The enhanced anti-corrosion properties of graphitized carbon substrates are evaluated via accelerated stress tests (ASTs). The graphitized surface properties of carbon are responsible for alleviating the coarsening and agglomeration of Pt particles.

## Graphical abstract

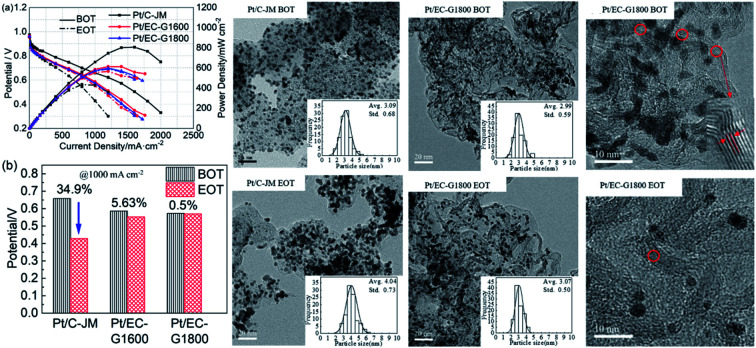

## 1. Introduction

Polymer electrolyte membrane fuel cells (PEMFCs) are regarded as a promising, clean power source for automobiles due to their high efficiency, high output power density, low operation temperature, and near-zero emissions.^1–5^ Recently, Pt and Pt-based ultrasmall particles supported on carbon matrices have shown state-of-the-art performance toward the oxygen reduction reaction (ORR).^6–8^ However, durability is still one of the major obstacles preventing the commercialization of PEMFCs.^9–12^ There are numerous studies on the degradation of cathode catalysts; in these, Pt particle dissolution and agglomeration, and carbon support corrosion are considered the main reasons for this degradation.^13–17^ Thus, the stability and anti-corrosion properties of carbon supports for cathode catalysts play a vital role in the commercialization of PEMFCs.

For automobile applications, the local cathode potential can reach as high as 1.5 V during starting and stopping due to the H_2_/air frontier, which accelerates the oxidization of the carbon support and speeds up the degradation of the PEMFC.^18,19^ When carbon oxidation occurs, oxygen-containing functional groups are generated on the surface of the carbon support, weakening the interactions between the platinum particles and the carbon support. The platinum particles will migrate and agglomerate, finally detaching from the carbon support.^20,21^ The interfacial structure formed between the Pt nanoparticles and carbon support changes because of the functional groups generated on the carbon surface.^22,23^ The electrons in Pt nanoparticles near the oxygen functional groups can be transferred to the electronegative oxygen atoms, increasing the oxidation state of Pt; this can trigger the easy dissolution of Pt nanoparticles and accelerate the Ostwald ripening process.^24,25^ Meanwhile, the conductivity of the carbon support decreases; as a result, the impedance of the fuel cell increases.^20^ In addition, the carbon support will become hydrophilic, which can have an impact on gas permeability as well as water management. Therefore, high constant potential or potential cycling tests are usually conducted as protocols to investigate the effects of carbon support corrosion on PEMFC performance. Protocols proposed by the US Fuel Cell Council (USFCC),^19^ US Department of Energy (DOE),^26^ and the Fuel Cell Commercialization Conference of Japan (FCCJ)^27–29^ are widely used as standard protocols. Potential undulation during the start-stop protocol can be simulated *via* potential cycling to accelerate the degradation of the catalyst support within a short period of time.^30,31^

To address the above-mentioned stability issues affecting carbon carriers, a lot of effort has been made to improve the inherent properties of ORR catalysts; in these studies, substitution and surface modification and coating are mainly employed.^32,33^ Jung *et al.* employed graphitized carbon black and a N-doped graphitic carbon matrix as supports to increase corrosion resistance and to introduce N-modified active sites onto the carbon surface.^34,35^ Chen developed nitrogen/metal co-doped graphene tubes as substrates for stable ORR catalysts.^36^ Highly graphitized carbon materials, such as carbon nanofibers and carbon nanotubes, have been employed due to their unique corrosion resistance properties. However, the chemically inert planes of carbon nanotubes lead to low Pt nanoparticle dispersibility, large crystallite/particle sizes, and ease of agglomeration, resulting in the rapid and massive loss of the electrochemically active surface area (ECSA).^37,38^ It has been observed that the agglomeration of Pt nanoparticles mainly occurs at the junctions and edges of graphited carbon particles, based on electron tomography.^39^ Therefore, determining the degree of graphitization of the carbon support appears to require an optimum balance between uniform metal-particle dispersion and sufficient carbon corrosion resistance in order to meet the requirements of the various operating conditions of automotive PEMFCs, especially operation conditions that cause high potentials.

In this work, the degree of graphitization of the carbon support was adjusted, and the resulting materials were used to synthesize platinum-based catalysts. The durability of single cells with the prepared catalysts located at the cathode was examined using accelerated stress test (AST) protocols, including under potential-cycling simulated start-stop and high potential holding conditions. The structural changes in the carbon supports and Pt nanoparticles were monitored *via* transmission electron microscopy (TEM).

## 2. Experiments and methods

### 2.1. Carbon black graphitization

The degree of graphitization of carbon black (Ketjenblack EC-600JD, Akzo Nobel N.V) was increased *via* heat treatment under N_2_ protection (>99.99%, Air Chemical Co., Ltd). KetjenBlack EC-600JD was thoroughly premixed with Ni(NO_3_)_2_ (Sinopharm Chemical Reagent Co., Ltd) before heat treatment to promote the graphitization reaction of carbon black and reduce the temperature required for graphitization. The details are as follows. Appropriate amounts of EC-600JD and Ni(NO_3_)_2_, based on a C : Ni mass ratio of 20 : 1, were evenly dispersed in ultrapure water (18.2 MΩ cm at 25 °C) *via* ultrasonic vibration. The well-mixed suspension was transferred to a rotary evaporator (Shanghai Dongxi Refrigeration Equipment Co., Ltd) and subjected to treatment at 100 °C to obtain dried powder. The powder was then heat-treated for 3 h at a predetermined temperature (1600 °C or 1800 °C) at a heating rate of 20 °C min^−1^ in a vacuum furnace (Shanghai Chen Hua Technology Co., Ltd) under a N_2_ atmosphere. Finally, the heat-treated powder was washed, filtered, dried, and labeled as EC-G1600 or EC-G1800, depending on the treatment temperature.

### 2.2. Catalyst synthesis

The catalysts supported on the as-prepared graphitized carbon black samples, *i.e.*, Pt/EC-*x* (where *x* is G1600 or G1800), were synthesized *via* the one-pot ethylene glycol (Sinopharm Chemical Reagent Co., Ltd) reduction of the Pt precursor H_2_PtCl_6_·6H_2_O (Sinopharm Chemical Reagent Co., Ltd). The pH value of the ethylene glycol solution was adjusted to 12 with NaOH (Sinopharm Chemical Reagent Co., Ltd) before being heated to 130 °C under a N_2_ atmosphere and maintained for 3 h. After cooling to room temperature, the obtained solid was filtered with abundant ultrapure water and dried in a vacuum oven for 12 h. For comparison, Pt supported on raw carbon black (EC-600JD) was prepared using the same method and labelled as Pt/EC. Commercial Pt/C (60 wt% Pt, Johnson Matthey) was used as received and labelled as Pt/C-JM.

### 2.3. Physical characterization

TEM analysis (JEM-2100F, Nippon Electronics Co., Ltd) was conducted at 200 kV to observe the fresh Pt/C catalysts and aged Pt/C catalysts that underwent durability testing. Raman spectroscopy (LabRAM HR Evolution, HORIBA Scientific) analysis was carried out with radiation from an argon laser (514 nm) to examine the structures of the raw and graphitized carbon black samples. Specific surface area and porosity measurements using all carbon black samples were performed with a volumetric device (ASAP 2000, Micromeritics). X-ray diffraction (XRD, D8 ADVANCE, Bruker AXS) was used with Cu Kα radiation to examine the as-prepared Pt-based catalysts.

### 2.4. Electrochemical tests

Electrochemical tests were carried out with a CHI660D potentiostat (CH Instruments). A glassy carbon rotating disk (diameter: 5.6 mm; area: 0.246 cm^2^) coated with the corresponding catalyst was used as the working electrode. A reversible hydrogen electrode (RHE) and Pt wire were used as the reference and counter electrodes, respectively.

Homogeneous catalyst ink was prepared as follows. 2 mg of the studied catalyst was dispersed in 1 mL of a mixed solvent containing isopropanol (Sinopharm Chemical Reagent Co., Ltd) and an ionomer (Nafion, 5 wt%, DuPont) (the mass ratio was 30 : 1), and this was sonicated for 30 min. 10 μL of ink was transferred onto the rotating disk. The cyclic voltammetry (CV) technique was used to determine the electrochemically active surface area (ECSA) *via* integrating the hydrogen desorption charge from CV at a rate of 100 mV s^−1^ in N_2_-saturated 0.1 M HClO_4_ solution. The formula is as follows: ECSA = *Q*/(*m* × *k*), where *Q* is the integral charge of the H desorption peak after removing the baseline of the double-layer area, *m* is the mass of Pt loaded on the working electrode, and *k* is equal to a constant of 0.21 mC cm^−2^, which represents the average amount of charge for removing a single layer of H atoms from a pure Pt surface. The current was normalized to the Pt amount to get the mass activity.

### 2.5. Single-cell tests

Single-cell tests were conducted using a fuel cell test bench (G20, Greenlight Innovation). Membrane electrode assemblies (MEAs) were prepared *via* spraying catalyst ink onto Nafion® 212 membranes (DuPont) with the heating platform set at 80 °C, and then one piece of gas diffusion layer (SGL, 24BC) was physically placed on each side of the catalyst-coated membrane. The Pt loading of the cathode catalyst layer is 0.4 mg_Pt_ cm^−2^. The cathode catalyst ink was obtained *via* dispersing the studied catalyst into a mixed solvent made up of ultrapure water, isopropanol, and an ionomer dispersion (Nafion, 5 wt%, DuPont), and this was sonicated for 30 min. The ratio of catalyst to Nafion® ionomer was 3 : 1. All anode catalyst layers were prepared using commercial Pt/C-JM with the same method as the cathode and with Pt loading of 0.2 mg_Pt_ cm^−2^. The active electrode area of the obtained single cell is 25 cm^2^.

During the polarization curve testing of single cells, pure hydrogen and compressed air were supplied to the anode and cathode sides with stoichiometric ratios of 1.5 (anode) and 3.8 (cathode); a backpressure of 100 kPa and relative humidity (RH) of 80% were employed at both sides. The temperature of the cell was maintained at 80 °C using a water thermostat.

### 2.6. AST protocols

**Fig. 1:**
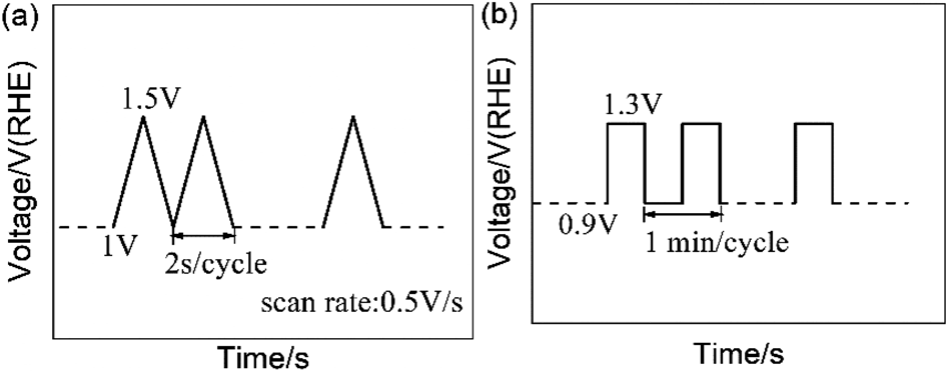
Accelerated stress test (AST) protocols: (a) the RDE test protocol and (b) the simulated start-stop protocol for single-cell testing.

The carbon-corrosion effects and degradation behaviors of the catalysts were scrutinized *via* employing AST protocols. For electrochemical environment testing, triangle wave potential cycling between 1 and 1.5 V *vs.* RHE with a sweep rate of 500 mV s^−1^ was employed to simulate the high potential conditions a fuel cell stack may encounter.^40,41^ For single-cell testing, a square wave between 0.9 V and 1.3 V (*vs.* anode) with a frequency of 1 min per cycle was used to simulate the conditions experienced by a MEA during start-stop operations.^30,31^ The AST protocols are shown in Fig. 1. In addition, potentiostatic AST testing was performed *via* holding the cathode potential at 1.4 V for 30 min.^42^

## 3. Results and discussion

### 3.1. Characterization of prepared carbon black and catalyst samples

#### 3.1.1. XRD and Raman analysis

**Fig. 2:**
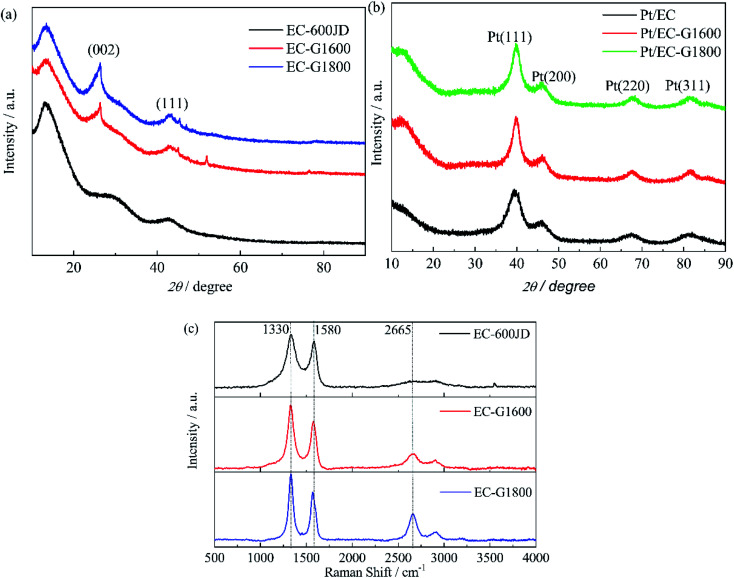
The XRD patterns of (a) EC-600JD (black), EC-G1600 (red), and EC-G1800 (blue), and (b) Pt/EC (black), Pt/EC-G1600 (red), and Pt/EC-G1800 (green). (c) The Raman spectra of EC-600JD (black), EC-G1600 (red), and EC-G1800 (blue).

The crystallinities and structures of the EC-600JD raw material and graphitized products were first inspected using XRD, as shown in Fig. 2(a). The peaks observed at 2*θ* values of 25° and 43° are assigned to the (002) and (101) diffraction planes of graphitic carbon, respectively.^43^ Compared to the clear patterns of the graphitized samples (EC-G1600 and EC-G1800), the signals of the raw material (EC-600JD) are immensely broadened, revealing the presence of large amounts of amorphous carbon in the material.^44^ The graphitic reflections become more legible after graphitizing, and they become even more well-resolved as the graphitization temperature rises. Raman spectroscopy is an available analytical tool for researching the inherent properties of carbonaceous materials. As shown in Fig. 2(c), the EC-600JD raw material and its graphitized products exhibit typical prominent broad peaks: a D peak (∼1330 cm^−1^) and a G (∼1580 cm^−1^) peak. The D-to-G peak intensity ratio (*I*_D_/*I*_G_) is merely 1.09 for EC-600JD, but it is effectively strengthened to 1.28 for EC-G1600 and further to 1.37 for EC-G1800. The value of *I*_D_/*I*_G_ displays an overall tendency to increase from the raw material EC-600JD to the final two graphitized carbon products. According to the literature,^45^ the *I*_D_/*I*_G_ ratio from the Raman spectrum depends on the amorphization trajectory, ranging from perfect graphite sheets to highly amorphous sp^3^ carbon; this consists of three stages: (1) from graphite to nanocrystalline graphite; (2) from nanocrystalline graphite to a-C; and (3) from a-C to ta-C.

In stage (1), *I*_D_/*I*_G_ increases according to the TK equation:^46^
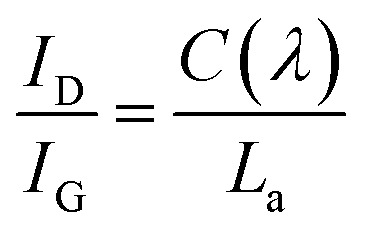
where *C* (515.5 nm) is ∼44 Å and *L*_a_ is the cluster diameter or in-plane correlation length. In stage (2), defects are gradually introduced into the graphite layer. The end of this stage corresponds to complete disorder. *I*_D_/*I*_G_ decreases with increasing amorphization. In stage (3), *I*_D_/*I*_G_ is very low or 0. During the graphitization process, some of the amorphous state progressively transforms into nanocrystalline graphite. Defects inevitably exist in the graphite layer of the final graphitized carbon black product. Therefore, the amorphization trajectory for all samples are in stage (2). *I*_D_/*I*_G_ will increase with decreasing disorder. Thus, a higher *I*_D_/*I*_G_ ratio for graphitized carbon means less disorder in the graphitic bonds.

#### 3.1.2. Isothermal nitrogen adsorption and desorption

**Fig. 3:**
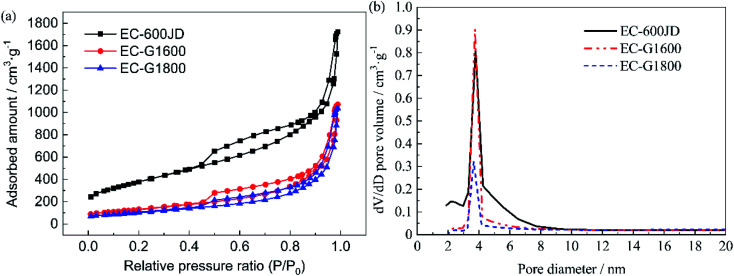
(a) The isothermal nitrogen adsorption and desorption curves and (b) pore volume distributions of the carbon supports.

Fig. 3(a) shows the isothermal nitrogen adsorption and desorption curves of the original EC600JD carbon black sample and the materials graphitized at different temperatures (EC-G1600 and EC-G1800). These are type-IV curves, based on the International Union of Pure and Applied Chemistry (IUPAC) classification.^47^ The response in the relative pressure (*P*/*P*_0_) range from 0 to 0.98 consists of three stages: low-pressure (0–0.03); medium-pressure (0.03–0.86); and high-pressure (0.86–0.98) areas. The micropores are mainly filled in the low-pressure area. Compared with untreated EC-600JD, the slopes of the absorption–desorption curves of EC-G1600 and EC-G1800 in this region decrease, which indicates that the microporous structure of the graphitized carbon is reduced significantly. In the medium-pressure region, the hysteresis loops caused by the capillary condensation of nitrogen molecules reveal the mesoporous structures of all the samples.^48^ In the high-pressure region, the hysteresis loops of the three types of carbon support reveal the existence of mesoporous structures. All of the carbon supports are dominated by mesoporous structures. The Brunauer–Emmett–Teller (BET) surface areas of the three samples were calculated to be 1360 m^2^ g^−1^, 634 m^2^ g^−1^, and 458 m^2^ g^−1^ for EC-600JD, EC-G1600, and EC-G1800, respectively. The pore volume distributions were evaluated *via* the Barrett–Joyner–Halenda (BJH) method, and they are provided in Fig. 3(b). EC-600JD contains a large number of microporous and mesoporous structures. After graphitization treatment, the pore size distribution is concentrated in the form of mesopores with a diameter of 3–5 nm, with the elimination of microporous structures. The BET specific surface areas of the graphitized materials (EC-G1600 and EC-G1800) dropped by more than 50% compared to that of raw carbon black (EC-600JD). This agrees with the adsorption–desorption curve and pore volume distribution curve results for the samples.

#### 3.1.3. XRD studies of the prepared catalysts

Fig. 2(b) provides the XRD patterns of the Pt/EC-*x* catalysts with the representative diffraction peaks. The corresponding crystal planes are marked in Fig. 2(b). The signals at ∼39.8°, ∼46.0°, ∼67.8°, and ∼81.7° can be indexed to the (111), (200), (220), and (311) crystal planes, respectively, of the face-centered cubic structure of Pt. It can be found that all of the as-prepared samples exhibit the four typical characteristic peaks of the face-centered cubic structure of Pt, indicating the fine degree of crystallization of the samples.

### 3.2. RDE testing of the prepared catalysts

**Fig. 4:**
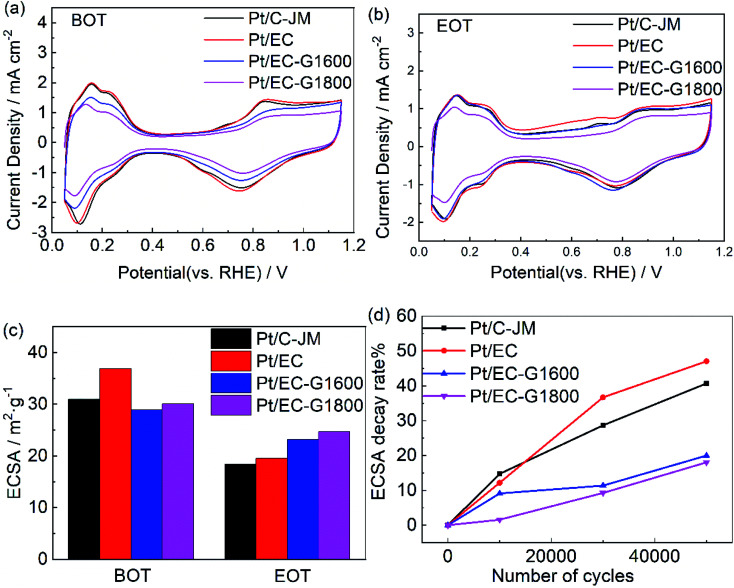
The H adsorption/desorption CV curves of Pt/EC (red), Pt/EC-G1600 (blue), Pt/EC-G1800 (magenta), and Pt/C (black) before (a) and after (b) 50 000 cycles. (c) The corresponding ECSA values of Pt/EC, Pt/EC-G1600, Pt/EC-G1800, and Pt/C before and after 5000 potential cycles from the CV data. (d) The corresponding ECSA decay rates of Pt/EC, Pt/EC-G1600, Pt/EC-G1800, and Pt/C from the CV data.

CV scans from the as-prepared catalysts obtained in N_2_-saturated 0.1 M HClO_4_ before and after 50 000 cycles are shown in Fig. 4(a) and (b), respectively. The typical H adsorption/desorption peaks are exhibited between 0.05 and 0.35 V (*vs.* RHE) in the CV curves. The ECSAs of each sample before and after ASTs were calculated using the process outlined in the Experimental section, and the results are summarized in Fig. 4(c). The initial ECSAs of Pt/EG-G1600 and Pt/EG-G1800 are roughly similar to commercial Pt/C-JM, which indicates that they possess fairly close available active surface areas for catalysis. However, Pt/EG-G1800 retains the highest ECSA after 50 000 cycles of potential scanning. Pt/EC exhibits the highest initial ECSA due to the high surface area of EC and the superior dispersion of Pt nanoparticles on its surface, which could be confirmed from TEM images (Fig. 7). However, Pt/EC shows the fastest degradation among the samples, which results from the surface oxygen functionalization of the carbon support. The higher ECSA but worse stability is consistent with previous reports.^25^ The corresponding ECSA decay levels, normalized to the initial ECSAs, of the catalysts as a function of the number of cycles are given in Fig. 4(d). After 50 000 cycles of stress, Pt/EC-G1800, Pt/EC-G1600, Pt/EG, and Pt/C-JM lost 18.0%, 20.0%, 47.0%, and 40.7% of their initial ECSAs, respectively. The decay rates of Pt/EC-G1600 and Pt/EC-G1800 are greatly reduced during the accelerated potential cycling. These degradation results show that graphitized carbon black supports can not only effectively inhibit the loss of the active area of catalyst nanoparticles but they can also improve the stability of Pt-based catalysts.

### 3.3. Single-cell testing of the prepared MEAs

#### 3.3.1. Simulated start-stop cycling

**Fig. 5:**
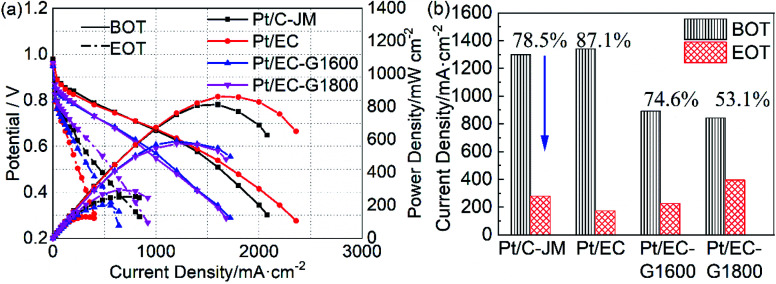
(a) The polarization and power density curves of Pt/EC (red), Pt/EC-G1600 (blue), Pt/EC-G1800 (magenta), and Pt/C-JM (black) before (BOT, solid lines) and after (EOT, dash-dotted lines) the start-stop protocol. (b) The decay rates of the current density at 0.6 V from the corresponding polarization curves of the four catalysts.

To simulate the operating environment of the start-stop conditions that PEMFCs are constantly subjected to during the actual operation of fuel cell vehicles, we used start-stop cycles to evaluate the durability of the cathode catalysts in PEMFCs. The initial polarization curves of single cells prepared with the four catalysts as cathode catalysts are shown in Fig. 5(a). Pt/C-JM and Pt/EC showed similar initial performances. The Pt/EC-G1600 and Pt/EC-G1800 catalysts exhibited comparable initial performances, slightly lower than the catalysts with non-graphitized carbon supports. It is worth noting that after 1000 cycles of start-stop accelerated cycle testing, the performances of Pt/C-JM and Pt/EC are drastically lower than those of the graphitized carbon carriers. Measured before and after ASTs, the corresponding current density and decay rate values at 0.6 V are shown in Fig. 5(b). The decay rates of the Pt/C-JM and Pt/EC single cells are as high as 78.5% and 87.1%, respectively. The Pt/EC-G1800 fuel cell showed the highest current density after accelerated testing, and the decay rate was the lowest, 53.1%, which indicates that the Pt/EC-G1800 catalyst maintains the maximum catalytic activity and the best performance after start-stop cycle tests.

#### 3.3.2. High potential protocol

TEM images of the catalysts before and after the start-stop AST protocol are exhibited in Fig. 7(a–h). Before AST testing, the platinum particles of the four catalysts have broadly the same particle size, about 3 nm. After start-stop cycle testing, the Pt particles of the Pt/C-JM and Pt/EC catalysts showed severe Ostwald ripening and significant coarsening was observed. The morphology changes of the Pt/EC-G1600 and Pt/EC-G1800 catalysts are small, which is consistent with the performance degradation trends seen in the polarization curves in Fig. 5. The levels of growth of the Pt particles in the Pt/C-JM, Pt/EC, Pt/EC-G1600, and Pt/EC-G1800 catalysts are 1.57 nm, 2.51 nm, 0.98 nm, and 0.66 nm, respectively, as listed in Table 1. During the graphitization of carbon black, the oxygen functional groups on the carbon surface are gradually eliminated. Therefore, the Pt nanoparticles supported on the graphitized carbon support have a higher Pt^0^ phase content because of the existence of fewer oxygen functional groups on the carbon surface, resulting in more electrochemically stable Pt nanoparticles. On the surface of untreated carbon black, the electrons of the Pt nanoparticles near the oxygen functional groups migrate toward the negative oxygen atoms, leading to an increased level of Pt nanoparticles in an oxidized state, triggering ready dissolution and Ostwald ripening.^25^ It can be observed from the high-resolution TEM images of the Pt/EC-G1600 and Pt/EC-G1800 catalysts before and after start-stop ASTs in Fig. 7(l–o) that there are layered ordered lattice fringes on the carbon supports of the two catalysts before testing. The lattice spacing of 0.341 nm, measured using DigitalMicrograph, is consistent with the layer spacing of graphite.^49^ After start-stop ASTs, the graphite layer structure of the Pt/EC-G1600 catalyst carrier had mostly disappeared. The Pt/EC-G1800 carbon catalyst carrier retained its intact layer structure. The results indicate that the carbon support of the Pt/EC-G1600 catalyst begins to undergo gradual oxidation during the start-stop protocol, resulting in the gradual destruction of the graphite lattice structure. The carbon carrier of Pt/EC-G1800 has a complete graphite lattice structure, with the oxidation resistance being improved. It is indicated that the performance degradation of the cathode catalyst mainly results from the coarsening of the platinum particles, which is caused by the corrosion of the carbon support during start-stop cycling. After the graphitization of the carbon support, the growth of the platinum particles is effectively suppressed. Generally, the carbon supports of cathode catalysts are extremely corroded as a result of start-stop cycling, causing the pronounced detachment and aggregation of the Pt nanocrystallites.^50^ For Pt/C-JM, start-stop cycling resulted in the preferential oxidation of the chaotic domains of the carbon support. However, in the case of the graphitized carbon supports, the graphite lattice inhibits the oxidation process.

**Table 1:** The performance parameters before and after ASTs

	RDE testing	After start/stop protocol stress		After holding at 1.4 V for 30 min	
	Decay ratio of the ECSA/%	Decay ratio of the current density at 0.6 V/%	Growth of Pt particles/nm	Decay ratio of the potential at 1000 mA cm^−2^/%	Growth of Pt particles/nm
Pt/C-JM	38.7	78.5	1.57	34.9	0.95
Pt/EC	36.3	87.1	2.51	—	—
Pt/EC-G1600	19.0	74.6	0.98	5.63	0.17
Pt/EC-G1800	18.2	53.1	0.66	0.50	0.08

**Fig. 6:**
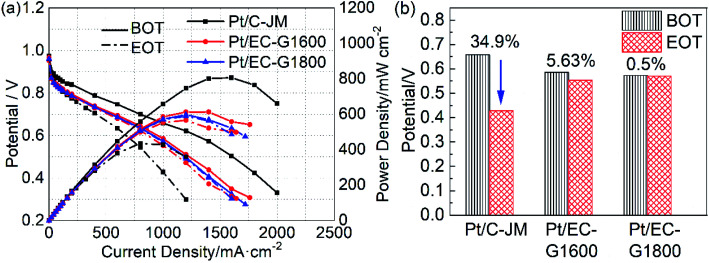
(a) The polarization and power density curves of Pt/EC-G1600 (red), Pt/EC-G1800 (blue), and Pt/C (black) before (BOT, solid lines) and after (EOT, dash-dotted lines) the high potential protocol. (b) The decay rates of the voltage at 1000 mA cm^−2^ from the corresponding polarization curves of the catalysts.

The high potential is at the root of the oxidation of carbon carriers. High potential operation tests were further conducted to evaluate the durability of the single cells. The polarization curves of the single cells before and after high-potential ASTs are provided in Fig. 6(a). Initially, the single-cell performances of the two catalysts with graphitized carbon supports were slightly lower than that of commercial Pt/C-JM. After high-potential ASTs, the performance of Pt/C-JM is much poorer than those of the as-prepared catalysts with graphitized carbon supports. The voltages corresponding to a current density of 1000 mA cm^−2^ and the decay rates are shown in Fig. 6(b). The decay rate of the Pt/C-JM single cell reached 34.9%, while the Pt/EC-G1800 fuel cell only declined by 0.50%, as listed in Table 1, indicating that the carbon carrier of the Pt/C-JM catalyst undergoes a large amount of oxidation at high potential, resulting in a sharp decline in performance. The carbon support of the Pt/EC-G1800 catalyst with a graphitized structure has improved oxidation resistance, so the catalyst can withstand corrosion at high potentials and the performance remains high.

**Fig. 7:**
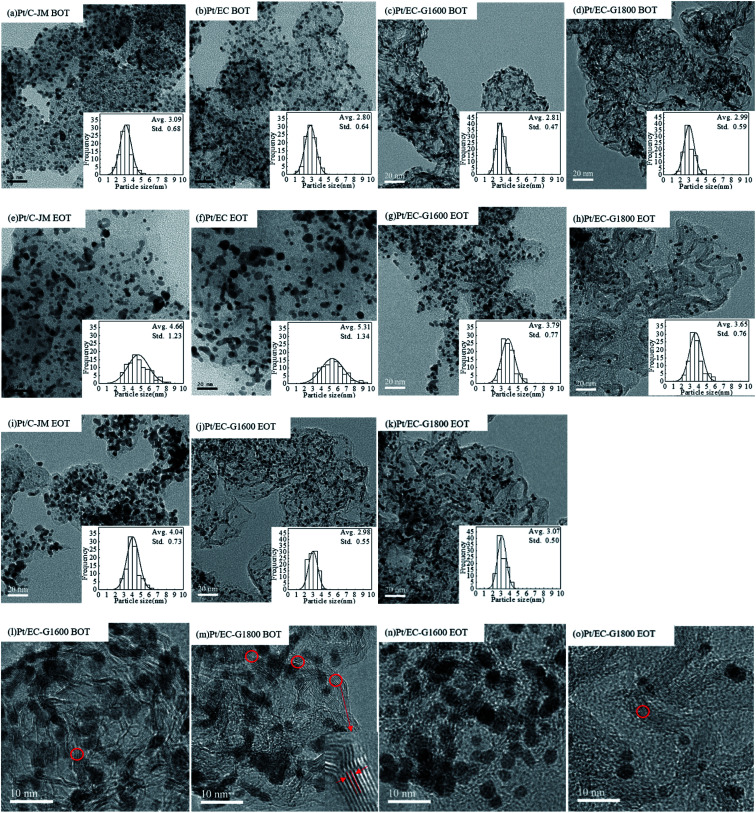
TEM images of samples: (a–d) samples before testing (BOT): Pt/C-JM (a), Pt/EC (b), Pt/EC-G1600 (c), and Pt/EC-G1800 (d); (e–h) samples at the end of start-stop protocol testing (EOT): Pt/C-JM (e), Pt/EC (f), Pt/EC-G1600 (g), and Pt/EC-G1800 (h); and (i–k) samples at the end of high potential protocol testing (EOT): Pt/C-JM (i), Pt/EC-G1600 (j), and Pt/EC-G1800 (k). (l–o) High-resolution TEM images of Pt/EC-G1600 (l) and Pt/EC-G1800 (m) before ASTs; and Pt/EC-G1600 (n) and Pt/EC-G1800 (o) after the start-stop AST protocol.

Similar to the morphologies of the catalysts during start-stop ASTs, the platinum particles of Pt/C-JM were severely coarsened, but the platinum particles supported on graphitized carbon did not significantly grow or coarsen after the high-potential AST protocol, as shown in Fig. 7(i–k). The Pt particles of the Pt/C-JM, Pt/EC-G1600, and Pt/EC-G1800 catalysts grew by 0.95 nm, 0.17 nm, and 0.08 nm, respectively, as listed in Table 1. At a high potential of 1.4 V, no significant oxidation occurs on the graphitized carbon supports, which results in no degradation in performance, as shown in the polarization curves in Fig. 6. The carbon oxidation kinetics are sensitive to the structure and proceed more quickly in disordered domains.^51^ During the high potential stress test protocol, the degradation of commercial Pt/C-JM with an amorphous carbon support structure was observed to be more rapid. Compared to the Pt/EC-G1600 catalyst, the carbon support of the Pt/EC-G1800 catalyst has a finer graphite lattice, so the kinetic oxidation rate is the lowest.

## 4. Conclusions

The degradation of as-prepared catalysts with graphitized carbon supports was investigated using both simulated start-stop cycling and high-potential AST protocols. The Pt/EC-G1800 catalyst with the carbon support with the most complete graphite lattice was more resistant to electrochemical corrosion than catalysts with a defective graphite lattice support (Pt/EC-G1600) or structurally disordered carbon supports (Pt/EC and Pt/C-JM) during both stress testing protocols.

During RDE testing, the Pt/EC-G1800 catalyst displayed the lowest amount of ECSA loss and the lowest attenuation rate, less than half of the decay rate of commercial Pt/C-JM (18.1% *vs.* 38.6%). According to the results following start-stop and high-potential AST protocols using single cells, the catalyst particles on the graphitized carbon supports underwent the smallest amounts of growth among all the samples. Meanwhile, the Pt/EC-G1800 catalyst maintained the maximum performance, with a decay rate of 0.50% compared to 34.9% for the Pt/C-JM catalyst, during high-potential ASTs. Moreover, the graphite lattice structure of its carbon support remained intact. During start-stop ASTs, the Pt particles of the Pt/EC-G1800 catalyst grew by 0.66 nm compared to 1.57 nm for commercial Pt/C-JM.

Therefore, using a graphitized carbon support can effectively improve the antioxidation capabilities, alleviating the coarsening and agglomeration of Pt nanoparticles. Graphitized carbon could act as a more stable catalyst support and offer an alternative solution under the demanding operating conditions faced by automotive PEMFCs.

## Conflicts of interest

No potential competing interests are reported by the authors.

## Supplementary Material
